# Supplementary material for: Modeling glioblastoma heterogeneity as a dynamic network of cell states
Source: Mol Syst Biol. 2021 Sep 16;17(9):e10105. doi: 10.15252/msb.202010105 (PMC8444284; doi:10.15252/msb.202010105)
Supplement: Supplementary file 6 — Source Data for Figure 5 [file MSB-17-e10105-s004.zip › Figure5A_sourcedata/GSEA_3071/hallmarks_stateV.GseaPreranked.1623742546329/HALLMARK_COAGULATION.html]

Details for gene set HALLMARK\_COAGULATION[GSEA]

|  || Dataset | state43071 |
| Phenotype | NoPhenotypeAvailable |
| Upregulated in class | na\_neg |
| GeneSet | HALLMARK\_COAGULATION |
| Enrichment Score (ES) | -0.54960155 |
| Normalized Enrichment Score (NES) | -2.6548793 |
| Nominal p-value | 0.0 |
| FDR q-value | 0.0 |
| FWER p-Value | 0.0 |
Table: GSEA Results Summary

  

Fig 1: Enrichment plot: HALLMARK\_COAGULATION      
 Profile of the Running ES Score & Positions of GeneSet Members on the Rank Ordered List

  

| PROBE | GENE SYMBOL | GENE\_TITLE | RANK IN GENE LIST | RANK METRIC SCORE | RUNNING ES | CORE ENRICHMENT || 1 | PDGFB |  |  | 283 | 1.080 | -0.0273 | No |
| 2 | DUSP6 |  |  | 408 | 0.944 | -0.0276 | No |
| 3 | CLU |  |  | 464 | 0.900 | -0.0158 | No |
| 4 | A2M |  |  | 532 | 0.846 | -0.0077 | No |
| 5 | PLAT |  |  | 651 | 0.773 | -0.0111 | No |
| 6 | CD9 |  |  | 738 | 0.728 | -0.0095 | No |
| 7 | C1R |  |  | 1696 | 0.437 | -0.1811 | No |
| 8 | USP11 |  |  | 1716 | 0.434 | -0.1740 | No |
| 9 | P2RY1 |  |  | 1798 | 0.418 | -0.1792 | No |
| 10 | PLAU |  |  | 1863 | 0.408 | -0.1813 | No |
| 11 | HTRA1 |  |  | 1958 | 0.395 | -0.1895 | No |
| 12 | CAPN5 |  |  | 2086 | 0.375 | -0.2044 | No |
| 13 | ANXA1 |  |  | 2165 | 0.366 | -0.2102 | No |
| 14 | ADAM9 |  |  | 2228 | 0.359 | -0.2132 | No |
| 15 | CRIP2 |  |  | 2419 | 0.332 | -0.2412 | No |
| 16 | CTSV |  |  | 2541 | 0.317 | -0.2564 | No |
| 17 | TIMP3 |  |  | 2826 | 0.286 | -0.3035 | No |
| 18 | MMP15 |  |  | 2885 | 0.282 | -0.3076 | No |
| 19 | PROS1 |  |  | 3097 | 0.260 | -0.3414 | No |
| 20 | MMP2 |  |  | 3234 | -0.257 | -0.3609 | No |
| 21 | WDR1 |  |  | 3447 | -0.289 | -0.3942 | No |
| 22 | ITGB3 |  |  | 3508 | -0.301 | -0.3982 | No |
| 23 | LTA4H |  |  | 3584 | -0.312 | -0.4048 | No |
| 24 | GSN |  |  | 3695 | -0.331 | -0.4176 | No |
| 25 | CTSO |  |  | 3774 | -0.344 | -0.4240 | No |
| 26 | MAFF |  |  | 3849 | -0.359 | -0.4292 | No |
| 27 | FURIN |  |  | 3861 | -0.361 | -0.4224 | No |
| 28 | CPQ |  |  | 3933 | -0.374 | -0.4267 | No |
| 29 | PEF1 |  |  | 4281 | -0.468 | -0.4812 | No |
| 30 | ARF4 |  |  | 4308 | -0.475 | -0.4745 | No |
| 31 | BMP1 |  |  | 4559 | -0.569 | -0.5081 | No |
| 32 | F2RL2 |  |  | 4778 | -0.697 | -0.5324 | Yes |
| 33 | S100A13 |  |  | 4791 | -0.705 | -0.5173 | Yes |
| 34 | DUSP14 |  |  | 4895 | -0.804 | -0.5170 | Yes |
| 35 | CTSB |  |  | 5007 | -0.948 | -0.5148 | Yes |
| 36 | SH2B2 |  |  | 5086 | -1.117 | -0.5021 | Yes |
| 37 | CAPN2 |  |  | 5088 | -1.120 | -0.4746 | Yes |
| 38 | CSRP1 |  |  | 5130 | -1.243 | -0.4517 | Yes |
| 39 | TIMP1 |  |  | 5134 | -1.251 | -0.4214 | Yes |
| 40 | MMP14 |  |  | 5146 | -1.297 | -0.3914 | Yes |
| 41 | SERPINE1 |  |  | 5161 | -1.377 | -0.3601 | Yes |
| 42 | PRSS23 |  |  | 5189 | -1.489 | -0.3285 | Yes |
| 43 | THBS1 |  |  | 5197 | -1.538 | -0.2918 | Yes |
| 44 | ITGA2 |  |  | 5201 | -1.557 | -0.2539 | Yes |
| 45 | LRP1 |  |  | 5234 | -1.801 | -0.2155 | Yes |
| 46 | F3 |  |  | 5242 | -1.927 | -0.1693 | Yes |
| 47 | CFH |  |  | 5257 | -2.141 | -0.1191 | Yes |
| 48 | FBN1 |  |  | 5263 | -2.234 | -0.0649 | Yes |
| 49 | FN1 |  |  | 5287 | -2.866 | 0.0015 | Yes |
Table: GSEA details [plain text format]

  

Fig 2: HALLMARK\_COAGULATION: Random ES distribution      
 Gene set null distribution of ES for **HALLMARK\_COAGULATION**
